# The effect of two intracavitary electrocardiogram guidance on the positioning and complications of peripherally inserted central catheters in newborns: A network meta-analysis

**DOI:** 10.1097/MD.0000000000044486

**Published:** 2025-09-12

**Authors:** Zhanglin Wang, Yan Gao, Yunhong Lei, Rong Zhang, Yi Zhang, Chaojin Zhao

**Affiliations:** a School of Nursing, Pingdingshan University, Pingdingshan, Henan Province, China; b Zibo Maternal and Child Health Hospital, Zibo, Shandong Province, China; c Yichang Hubo Medical Research Institute, Yichang, Hubei Province, China.

**Keywords:** catheter tip location, intracavitary electrocardiogram, network meta-analysis, newborns, peripherally inserted central catheter

## Abstract

**Background:**

Intracavitary electrocardiogram (IC-ECG) refers to the use of normal saline column or guidewire as a probe electrode to guide the electrocardiogram during central venous catheter-placement, and then determine the position of the catheter tip in real time according to the characteristic changes of the electrocardiogram P wave during the catheterization. However, there are still controversies regarding the accuracy of catheter tip positioning and the incidence of complications in guiding the placement of peripherally inserted central catheters (PICCs) in newborns using metallic guidewire IC-ECG and normal saline column IC-ECG. In this study, a network meta-analysis was used to evaluate the effects of these 2 methods on the accuracy of catheter tip positioning and the incidence of complications during neonatal PICC catheterization, providing evidence for their clinical application.

**Methods:**

Randomized controlled trials assessing IC-ECG‐guided PICC placement in newborns were collected up to May 30, 2025. Two evaluators independently screened the literature, extracted data, and assessed the risk of bias in the included studies. RevMan 5.3 and Stata14.0 software were used for statistical analysis.

**Results:**

A total of twenty studies were included, involving 2566 newborns. Network meta-analysis showed that metallic guidewire IC-ECG was optimal in the accuracy of tip position and the incidence of catheter-related bloodstream infections. Normal saline column IC-ECG had the lowest incidence of complications, phlebitis, arrhythmias, and thrombosis/occlusion.

**Conclusion:**

Metallic guidewire IC-ECG may be better than normal saline IC-ECG in PICC catheter tip location accuracy. In terms of complications, normal saline IC-ECG may have a lower risk.

## 1. Introduction

Peripherally inserted central catheter (PICC) has the characteristics of safety, reliability, hyperosmotic resistance and long retention time, and has been widely used in neonates with long-term infusion and intravenous nutrition support.^[1,2]^ The international standard recommends that the optimal tip position of a PICC catheter should be located within the lower one-third of the superior vena cava or at the junction between the superior vena cava and the upper wall of the right atrium.^[3]^ The accurate positioning of PICC catheter tip is important for reducing complications, prolonging catheter retention time, and ensuring the safety and best effect of intravenous therapy.^[4,5]^

Catheter tip ectopic placement, whether too deep or too shallow, can directly lead to complications.^[6]^ The tip position of the PICC catheter is too shallow, which increases the risk of phlebitis and thrombosis.^[4,7–9]^ If the PICC catheter tip is too deep, there is a risk of arrhythmia or heart cavity disease.^[10,11]^ At present, clinical methods to judge the tip of PICC include traditional anatomical landmark method, chest X-ray localization, intracavitary electrocardiogram (IC-ECG) localization, etc.^[12,13]^ For a long time, chest X-ray examination after PICC catheterization has been considered the “gold standard” for determining the position of the catheter tip.^[14]^ However, its lag delays the start time of infusion therapy in children and increases the harm of radiation exposure in children.^[15]^ The individual anatomy of newborns, especially preterm neonates, varies greatly. In addition, the position of the optimal catheter tip varies greatly with different gestational age and weight.^[16]^ As a result, it is difficult to determine the best tip position of PICC catheter accurately by traditional anatomical landmark method.

IC-ECG positioning technology is a practical technology to judge the real-time position of the catheter tip during PICC catheter-placement by observing the dynamic change of electrocardiogram waveform on the electrocardiogram monitor and adjust the catheter-placement depth in time to improve the one-time position rate of the catheter tip.^[4,17]^ A number of studies have shown that the safety and accuracy of IC-ECG is significantly higher than that of traditional localization methods such as anatomical landmark and chest X-ray.^[18–21]^ The Infusion Nurses Society recommends that IC-ECG be used to guide catheter tip positioning.^[22]^ European guidelines recommend real-time tip positioning in central venous catheters.^[23]^ This avoids the risk of catheter misalignment, reduces patient and medical staff exposure to X-rays, and saves time and costs.^[20]^

IC-ECG has been widely used in neonatal PICC catheterization.^[24,25]^ At present, the common guiding methods include metallic guidewire IC-ECG and normal saline column IC-ECG. Metallic guidewire IC-ECG involves inserting a metallic guidewire into the distal end of the catheter, and the proximal end is connected to the surface electrode through an electrocardiogram clip directly or indirectly through a converter. Normal saline column IC-ECG uses the conductivity of saline as a probe electrode to detect the change of electrocardiogram P wave and determine the position of catheter tip. It has the characteristics of short time, low cost, simple operation, safety and effectiveness.^[26]^ The above 2 methods can obtain intracavity electrocardiogram and determine the position of catheter tip through P wave change. In clinical practice, in order to avoid the damage of the guide wire to the intima, the guide wire must be extracted by 0.5 to 1.0 cm, which affects the judgment of the position of the catheter tip to a certain extent.^[27]^ Also, in the wire-guided mode, the extracted wire needs to be re-inserted into the catheter, increasing the risk of infection.^[27]^ Therefore, normal saline as electrical conduction should be the first choice for IC-ECG guided catheter tip location. Relevant studies have shown that it is recommended to avoid the use of metallic guidewire IC-ECG guidance for infants, and normal saline column IC-ECG guidance is safer for infants.^[28]^ Further clinical data support is needed for PICC catheterization guided by normal saline column IC-ECG in infants.

Metallic guidewire IC-ECG and normal saline column IC-ECG are the most commonly used guiding methods of IC-ECG, both of which have advantages and disadvantages. At present, the 2 methods lack direct comparative randomized controlled trials (RCTs) and cannot provide evidence support for clinical applications. The greatest advantage of network meta-analysis is that different interventions for the treatment of similar diseases can be summarized for quantitative statistical analysis and comparison, and the effect of a certain outcome indicator is ranked according to the quality, and then the optimal treatment plan is selected.^[29]^ Therefore, in order to better guide clinical practice, this network meta-analysis will compare the efficacy and safety of metallic guidewire IC-ECG and normal saline column IC-ECG in neonatal PICC catheter tip location, so as to provide a reliable decision basis for healthcare professionals.

## 2. Methods

### 2.1. Search strategy

A literature search was conducted on large databases including Cochrane Central Register of Controlled Trials, Web of Science, PubMed, Embase, CNKI, Wanfang database and SinoMed. RTCs assessing IC-ECG‐guided PICC placement in newborns were collected up to May 30, 2025. At the same time, references that have been included in the literature and related systematic reviews were tracked to obtain the literature that has not been retrieved. The search is carried out by the combination of MeSH term and free term, and the corresponding adjustment is made according to different databases. Search terms include the following: neonates, PICC, IC-ECG, EKG, ECG, catheter tip location, etc.

### 2.2. Literature inclusion and exclusion criteria

#### 2.2.1. Eligibility criteria

##### 2.2.1.1. Type of study

RCTs of neonatal PICC catheterization guided by IC-ECG.

##### 2.2.1.2. Types of participants

Neonatal with PICC catheterization during hospitalization.

##### 2.2.1.3. Types of interventions

The PICC was implanted using IC-ECG guidance (including metallic guidewire IC-ECG and normal saline column IC-ECG) or traditional anatomical landmark positioning, and the tube was placed under ultrasound guidance. X-ray is used to confirm location after tube placement.

##### 2.2.1.4. Types of outcomes

Primary outcomes: accuracy of catheter tip position and incidence of complications.

Secondary outcomes: incidence of phlebitis, catheter-related bloodstream infection (CRBSI), arrhythmias and thrombosis/occlusion.

#### 2.2.2. Exclusion criteria

1. Republished literature.
2. Comparison of different metal guides or different normal saline column methods.
3. Studies where data cannot be extracted.

### 2.3. Data extraction

Literature screening and data extraction were performed independently by 2 evaluators. Import references using Endnote and remove duplicate references. Evaluators conducted preliminary screening according to inclusion and exclusion criteria to extract available information. If there is a dispute, consult a third evaluator to discuss it, and contact the original author to resolve it if necessary. The main extraction contents included the first author, publication time, nationality, sample size, interventions, outcomes, etc.

### 2.4. Assessment of bias risk

The evaluation was conducted by 2 evaluators in accordance with the bias risk assessment tool provided by the Cochrane Handbook 5.1.0.^[30]^ Risk bias items include random sequence generation, allocation concealment, blinding study participants, blinding outcome evaluators, incomplete outcome data, selective reporting of results, and other biases. Each entry is classified as “low risk of bias,” “unclear,” “high risk of bias.”

### 2.5. Statistical analysis

Review Manager 5.3 software for direct comparison meta-analysis. Heterogeneity among included studies was analyzed by *I*^2^ test. *I*^2^ > 50% and *P* ≤ .1 indicated significant clinical heterogeneity, so a random effects model was selected for meta-analysis; On the contrary, a fixed effects model was used for meta-analysis. Relative risk (RR) and 95% confidence interval (CI) were used as statistical data. Sensitivity analysis was performed to detect the stability of meta-analysis results of primary outcome indicators by one-by-one exclusion method. We performed a frequency network meta-analysis by using Stata 14.0 software to analyze indirect comparisons between different interventions. In this study, there wass no closed loop, so the consistency model was adopted. The surface under the cumulative ranking (SUCRA) of various interventions was calculated, and the ranking map was drawn to obtain the probability of interventions with the best outcome indicators. The size of SUCRA was expressed as a percentage. The larger the SUCRA, the more effective the intervention or the lower the incidence of adverse reactions. Draw a comparison-adjusted funnel plot to determine whether there is publication bias.

## 3. Results

### 3.1. Study inclusion

**Figure 1.**
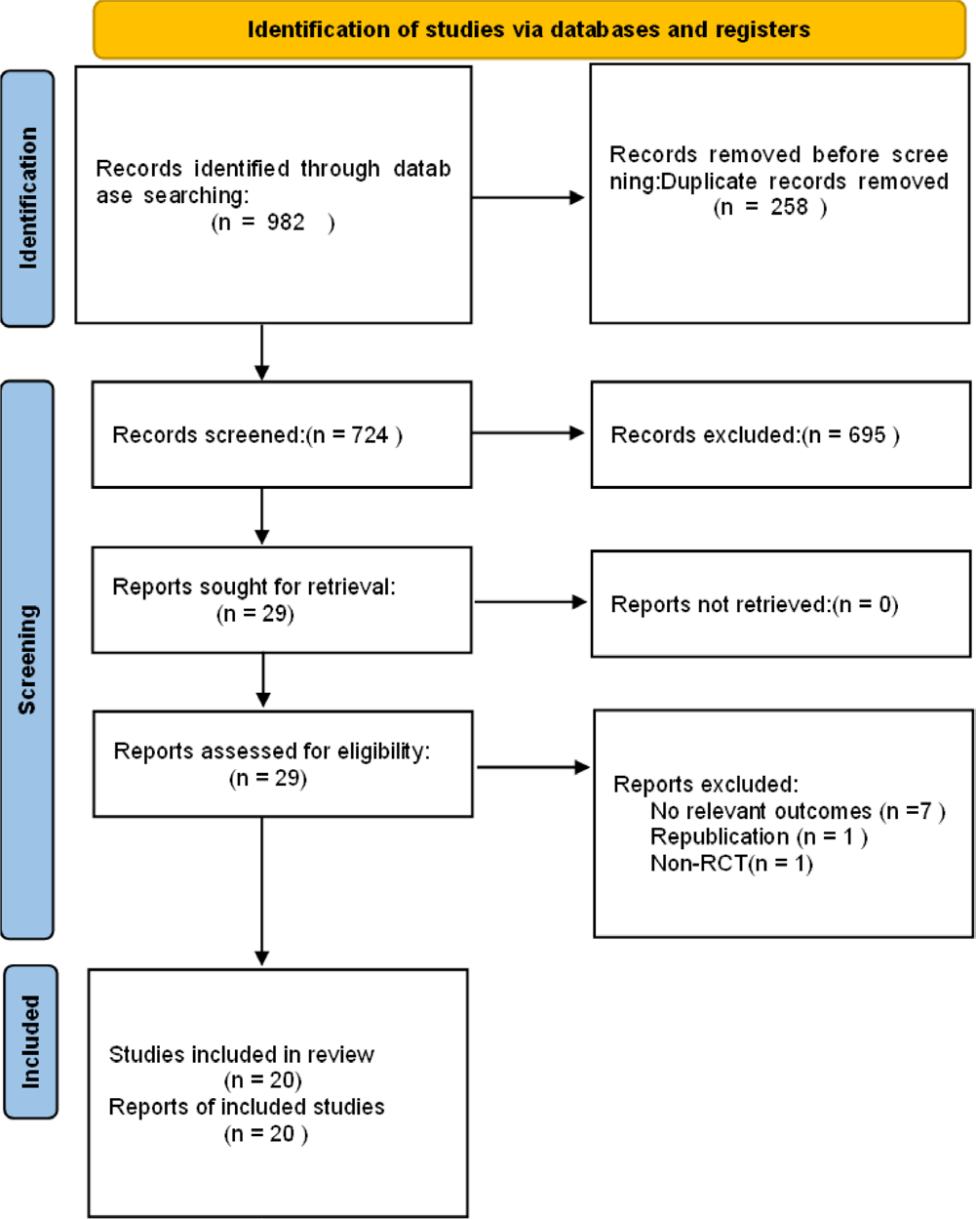
Flow chart of literature screening.

After layers of screening, a total of twenty studies^[7,15,28,31–47]^ were included, involving 2566 newborns (Fig. 1). The sample size of the included studies ranged from 42 to 210. The patient types were preterm neonates and term neonates. The catheter type used was 1.9Fr. The interventions of experimental group were metallic guidewire IC-ECG and normal saline column IC-ECG. The control group was placed under the guidance of ultrasound for traditional anatomical positioning. Lower limb veins were used in 3 studies. Superior vena cava was used in seventeen studies. The basic characteristics of the included studies are shown in Table 1.

**Table 1:** Basic characteristics of the included literature.

Study	Study location	Total patients	Study design	Type of patients	Catheters	Intervention		Approach
						Experimental group	Control group	
Ling et al,^[36]^ 2019	China	160	RCT	Preterm neonates	1.9Fr	Guide wire	Anatomical landmark	Superior vena cava
Jiang et al,^[34]^ 2021	China	172	RCT	Preterm neonates	1.9Fr	Normal saline	Anatomical landmark	Superior vena cava
Yu et al,^[46]^ 2021	China	130	RCT	Preterm neonates and term neonates	1.9Fr	Normal saline	Anatomical landmark	Superior vena cava
Tang et al,^[39]^ 2021	China	210	RCT	Term neonates	1.9Fr	Guide wire	Anatomical landmark	Superior vena cava
Huang T et al,^[32]^ 2020	China	62	RCT	Term neonates	NR	Normal saline	Anatomical landmark	Superior vena cava
Shi et al,^[38]^ 2019	China	148	RCT	Preterm neonates and term neonates	1.9Fr	Guide wire	Anatomical landmark	Superior vena cava
Yang C et al,^[42]^ 2021	China	180	RCT	Term neonates	NR	Guide wire	Anatomical landmark	Lower limb veins
Huang Y et al,^[33]^ 2020	China	144	RCT	Preterm neonates	1.9Fr	Normal saline	Anatomical landmark	Lower limb veins
Yang et al,^[43]^ 2023	China	184	RCT	Preterm neonates and term neonates	NR	Normal saline	Anatomical landmark	Superior vena cava
Wang et al,^[40]^ 2022	China	192	RCT	Preterm neonates	1.9Fr	Normal saline	Anatomical landmark	Superior vena cava
Yang 2022^[44]^	China	98	RCT	Preterm neonates and term neonates	NR	Normal saline	Anatomical landmark	Superior vena cava
Xiong et al,^[15]^ 2021	China	82	RCT	Preterm neonates and term neonates	1.9Fr	Guide wire	Anatomical landmark	Superior vena cava
Ling et al,^[7]^ 2022	China	120	RCT	Preterm neonates and term neonates	1.9Fr	Normal saline	Anatomical landmark	Lower limb veins
Liu et al,^[37]^ 2018	China	158	RCT	Preterm neonates	1.9Fr	Normal saline	Anatomical landmark	Superior vena cava
Zhu 2020^[47]^	China	94	RCT	Preterm neonates and term neonates	NR	Guide wire	Anatomical landmark	Superior vena cava
Wu et al,^[41]^ 2018	China	116	RCT	Preterm neonates and term neonates	1.9Fr	Guide wire	Anatomical landmark	Superior vena cava
Lin 2021^[35]^	China	60	RCT	Preterm neonates and term neonates	NR	Guide wire	Anatomical landmark	Superior vena cava
Hou 2018^[31]^	China	42	RCT	Preterm neonates and term neonates	1.9Fr	Normal saline	Anatomical landmark	Superior vena cava
Chen 2019^[28]^	China	160	RCT	Preterm neonates and term neonates	NR	Normal saline	Anatomical landmark	Superior vena cava
Yang et al,^[45]^ 2016	China	54	RCT	Preterm neonates	1.9Fr	Guide wire	Anatomical landmark	Superior vena cava

RCT = randomized controlled trial.

### 3.2. Quality of included studies

**Figure 2.**
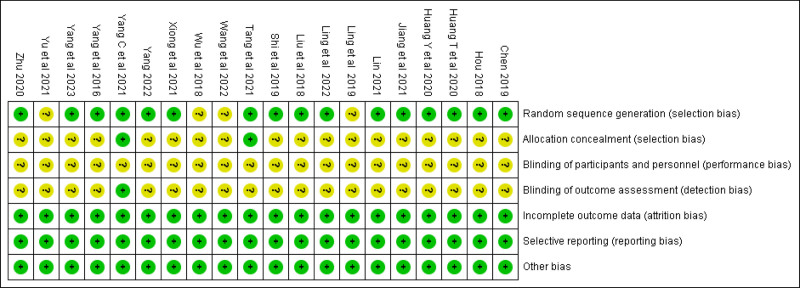
Quality assessment of included studies. Yellow (?): unclear risk; Green (+): low risk; Red (-): high risk.

A total of twenty RCTs were included, and the results of bias analysis were evaluated according to the Cochrane manual as follows (Fig. 2): all the twenty studies used random method to select the subjects. A total of sixteen studies described the generation of random order, while the remaining studies did not report which specific random allocation method was used. Two studies reported allocation concealment. One study reported the implementation of the blind method.

### 3.3. Pairwise meta-analysis

#### 3.3.1. Accuracy of catheter tip position

**Figure 3.**
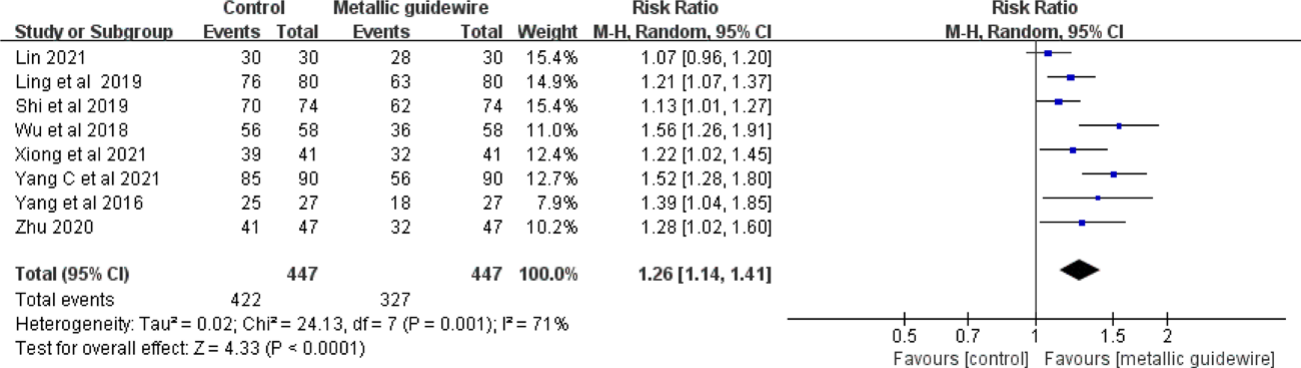
Forest plots of the tip position accurate (metallic guidewire vs anatomical landmark).

Eight studies evaluated the accuracy of metallic guidewire IC-ECG for PICC catheter tip position in neonates. The meta-analysis showed that the metallic guidewire IC-ECG method can improve the accuracy of PICC catheter tip position in neonates compared with traditional anatomical positioning (RR = 1.26, 95% CI (1.14, 1.41), *P* < .0001) (Fig. 3).

**Figure 4.**
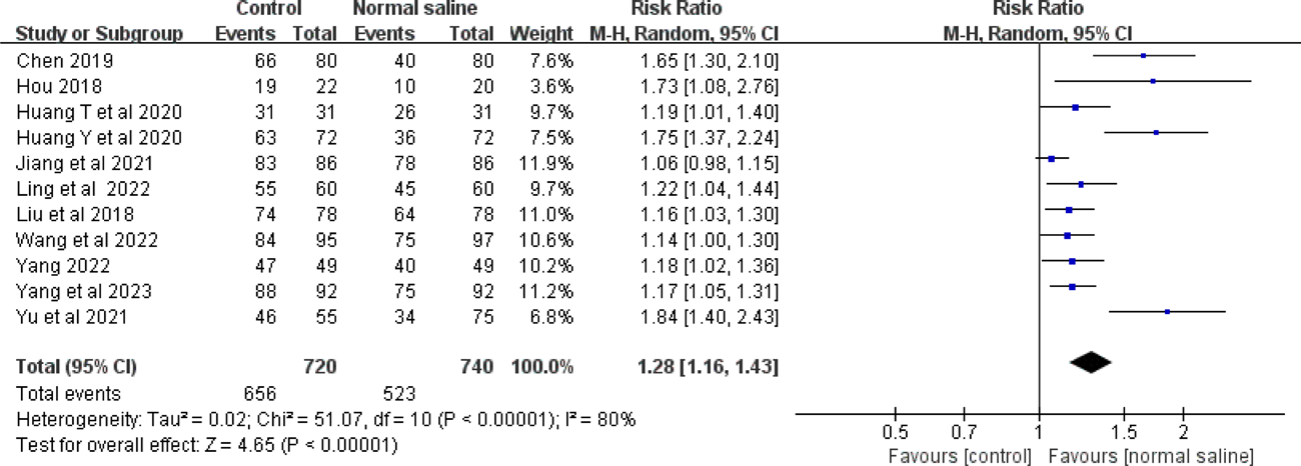
Forest plots of the tip position accurate (normal saline vs anatomical landmark).

Eleven studies evaluated the accuracy of normal saline column IC-ECG for PICC catheter tip position in neonates. The meta-analysis showed that normal saline column IC-ECG can improve the accuracy of PICC catheter tip position in neonates compared with traditional anatomical positioning (RR = 1.28, 95% CI (1.16, 1.43), *P* < .00001) (Fig. 4).

#### 3.3.2. Incidence of complications

**Figure 5.**
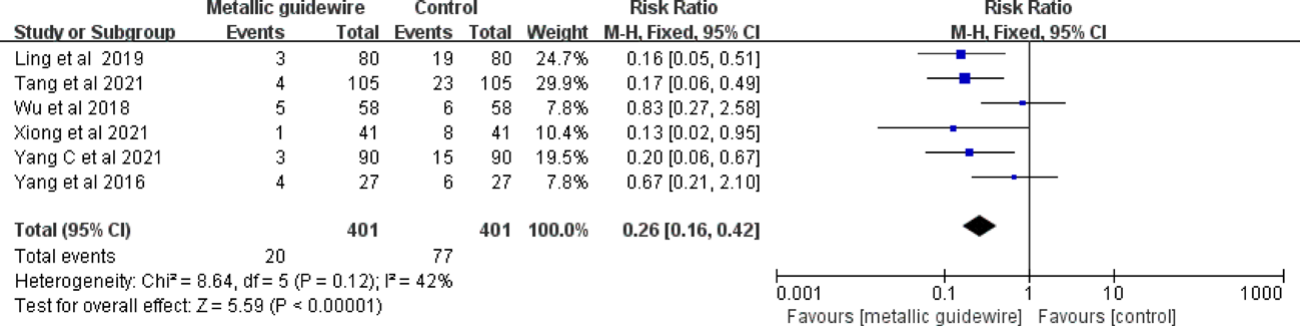
Forest plots of complication (metallic guidewire vs anatomical landmark).

Six studies evaluated the effect of metallic guidewire IC-ECG on the incidence of neonatal PICC catheter complications. The meta-analysis showed that the metallic guidewire IC-ECG method can reduce the incidence of neonatal PICC catheter complications compared with traditional anatomical positioning (RR = 0.26, 95% CI (0.16, 0.42), *P* < .00001) (Fig. 5).

**Figure 6.**
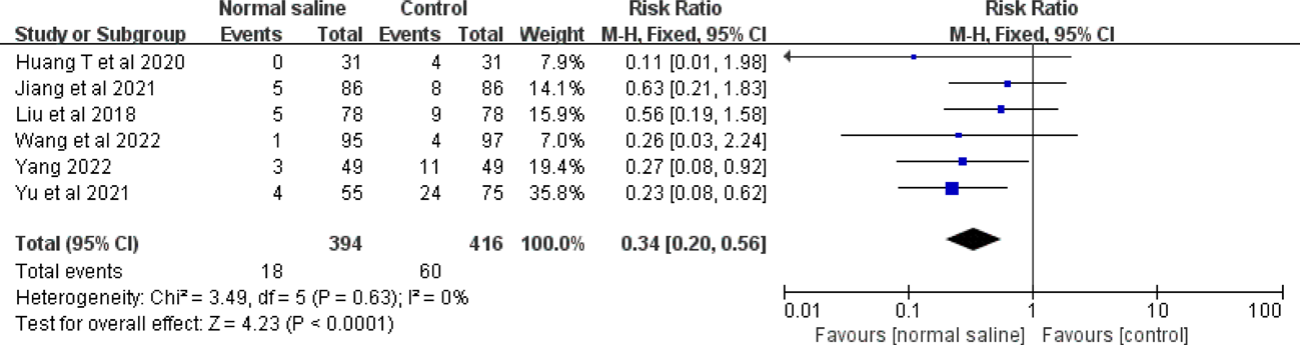
Forest plots of complication (normal saline vs anatomical landmark).

Six studies were conducted to evaluate the effect of normal saline column IC-ECG on the incidence of PICC catheterization in neonates. The meta-analysis showed that normal saline column IC-ECG can reduce the incidence of neonatal PICC catheter complications compared with traditional anatomical positioning (RR = 0.34, 95% CI (0.20, 0.56), *P*** **< .0001) (Fig. 6).

#### 3.3.3. Incidence of phlebitis

**Figure 7.**
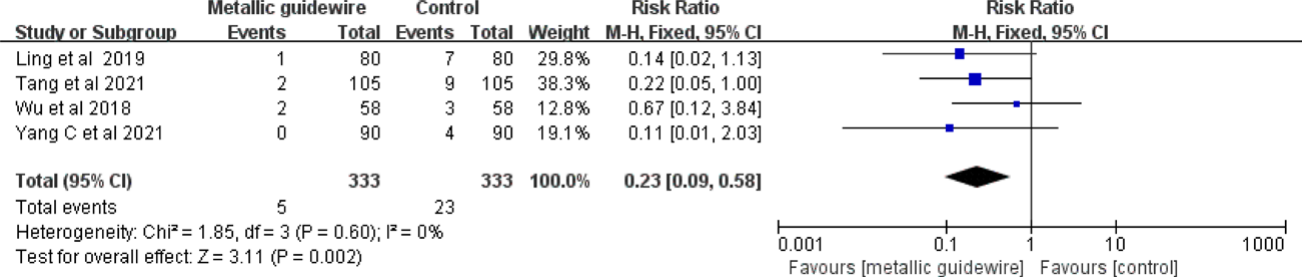
Forest plots of the incidence of phlebitis (metallic guidewire vs anatomical landmark).

Four studies evaluated the effect of metallic guidewire IC-ECG on the incidence of PICC-associated phlebitis in neonates. The meta-analysis showed that the metallic guidewire IC-ECG method reduced the incidence of neonatal PICC-associated phlebitis compared with traditional anatomical positioning (RR = 0.23, 95% CI (0.09, 0.58), *P* = .002) (Fig. 7).

**Figure 8.**
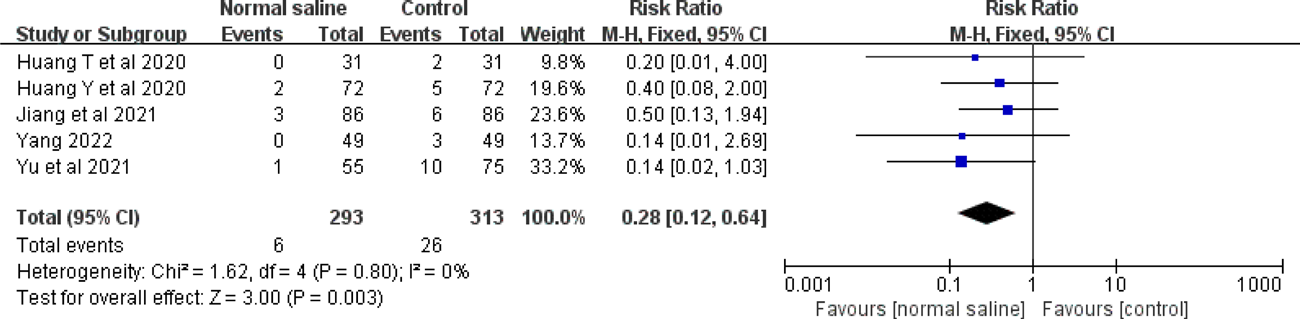
Forest plots of the incidence of phlebitis (normal saline vs anatomical landmark).

Five studies evaluated the effect of normal saline column IC-ECG on the incidence of PICC-associated phlebitis in neonates. The meta-analysis showed that the normal saline column IC-ECG method reduced the incidence of neonatal PICC-associated phlebitis compared with traditional anatomical positioning (RR = 0.28, 95% CI (0.12, 0.64), *P* = .003) (Fig. 8).

#### 3.3.4. Incidence of CRBSIs

**Figure 9.**
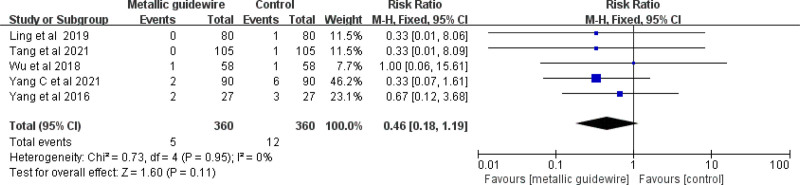
Forest plots of the incidence of catheter-related bloodstream infections (metallic guidewire vs anatomical landmark).

Five studies evaluated the effect of metallic guidewire IC-ECG on the incidence of neonatal CRBSIs. The meta-analysis showed no difference between traditional anatomical positioning and metallic guidewire IC-ECG in reducing the incidence of neonatal CRBSIs (RR = 0.46, 95% CI (0.81, 1.19), *P* = .11) (Fig. 9).

**Figure 10.**
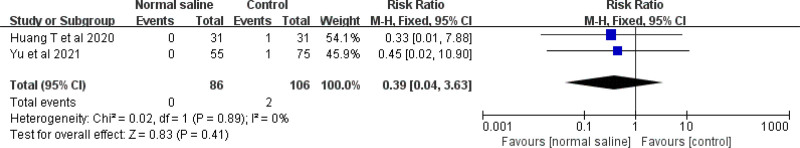
Forest plots of the incidence of catheter-related bloodstream infections (normal saline vs anatomical landmark).

Two studies evaluated the effect of normal saline column IC-ECG on the incidence of neonatal CRBSIs. The meta-analysis showed no difference between traditional anatomical positioning and normal saline column IC-ECG in reducing the incidence of neonatal CRBSIs (RR = 0.39, 95% CI (0.04, 3.63), *P* = .41) (Fig. 10).

#### 3.3.5. Incidence of arrhythmia

**Figure 11.**
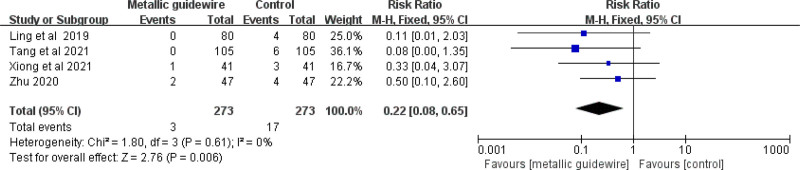
Forest plots of the incidence of arrhythmia (metallic guidewire vs anatomical landmark).

Four studies evaluated the effect of metallic guidewire IC-ECG on the incidence of arrhythmia. The meta-analysis showed that compared with traditional anatomical positioning, the metallic guidewire IC-ECG method can reduce the incidence of arrhythmia (RR = 0.22, 95% CI (0.08, 0.65), *P* = .002) (Fig. 11).

**Figure 12.**
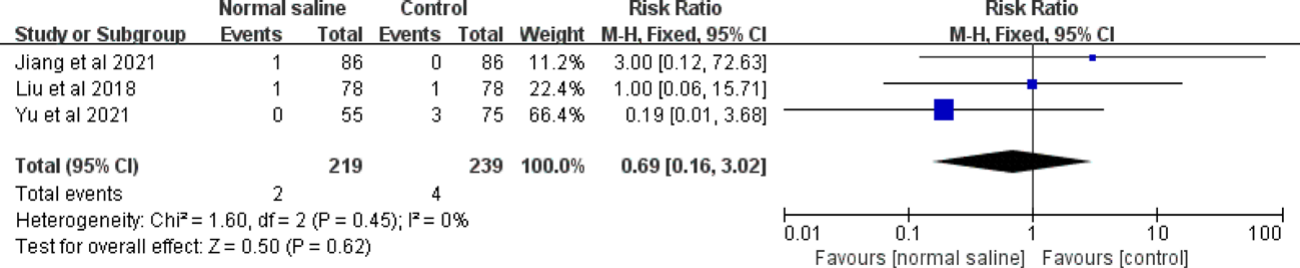
Forest plots of the incidence of arrhythmia (normal saline vs anatomical landmark).

Three studies evaluated the effect of normal saline column IC-ECG on the incidence of arrhythmia. The meta-analysis showed no difference between traditional anatomical positioning and normal saline column IC-ECG in reducing the incidence of arrhythmias in neonates (RR = 0.69, 95% CI (0.16, 3.02), *P* = .62) (Fig. 12).

#### 3.3.6. Incidence of thrombosis/occlusion

**Figure 13.**
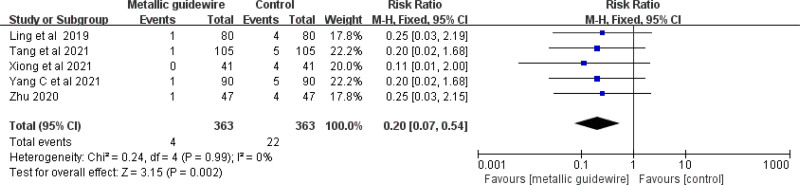
Forest plots of the incidence of thrombosis/occlusion (metallic guidewire vs anatomical landmark).

Five studies evaluated the effect of metallic guidewire IC-ECG on the incidence of thrombosis/occlusion in newborns. The meta-analysis showed that the metallic guidewire IC-ECG method reduced the incidence of thrombosis/occlusion in neonates compared with traditional anatomical positioning (RR = 0.20, 95% CI (0.07, 0.54), *P* = .002) (Fig. 13).

**Figure 14.**
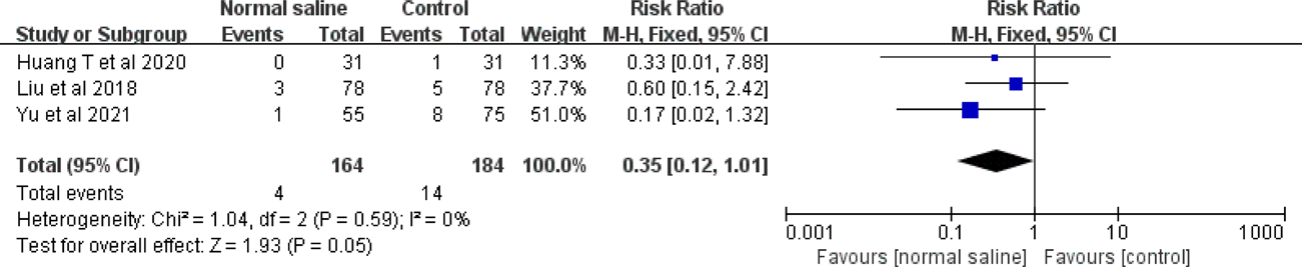
Forest plots of the incidence of thrombosis/occlusion (normal saline vs anatomical landmark).

Three studies evaluated the effect of normal saline column IC-ECG on the incidence of thrombosis/occlusion in neonates. The meta-analysis showed no difference between traditional anatomical positioning and normal saline column IC-ECG in reducing the incidence of thrombosis/occlusion in neonates (RR = 0.35, 95% CI (0.12, 1.01), *P* = .05) (Fig. 14).

### 3.4. Sensitivity analysis

**Figure 15.**
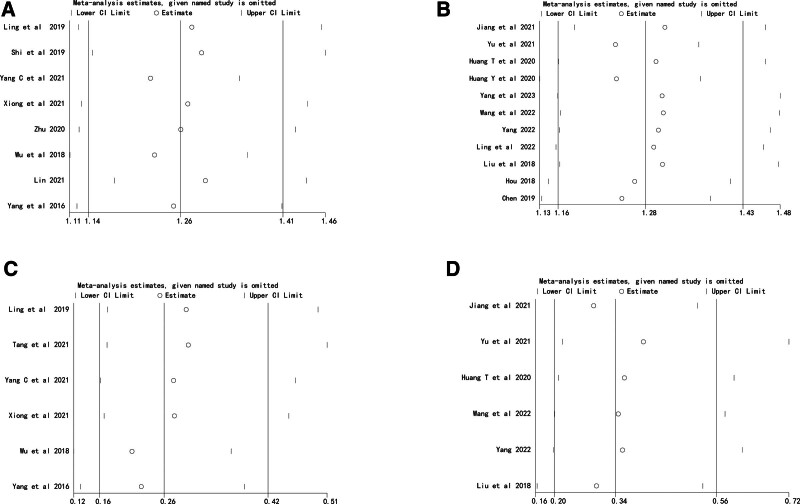
Sensitivity analysis. (A) Sensitivity analysis of the tip position accurate (metallic guidewire vs anatomical landmark). (B) Sensitivity analysis of the tip position accurate (normal saline vs anatomical landmark). (C) Sensitivity analysis of complication (metallic guidewire vs anatomical landmark). (D) Sensitivity analysis of complication (normal saline vs anatomical landmark).

We performed a sensitivity analysis of the results of a meta-analysis on the accuracy of catheter tip position and the incidence of complications. The results showed that the meta-analysis results were stable (Fig. 15).

### 3.5. Network meta-analysis

#### 3.5.1. Evidence network

**Figure 16.**
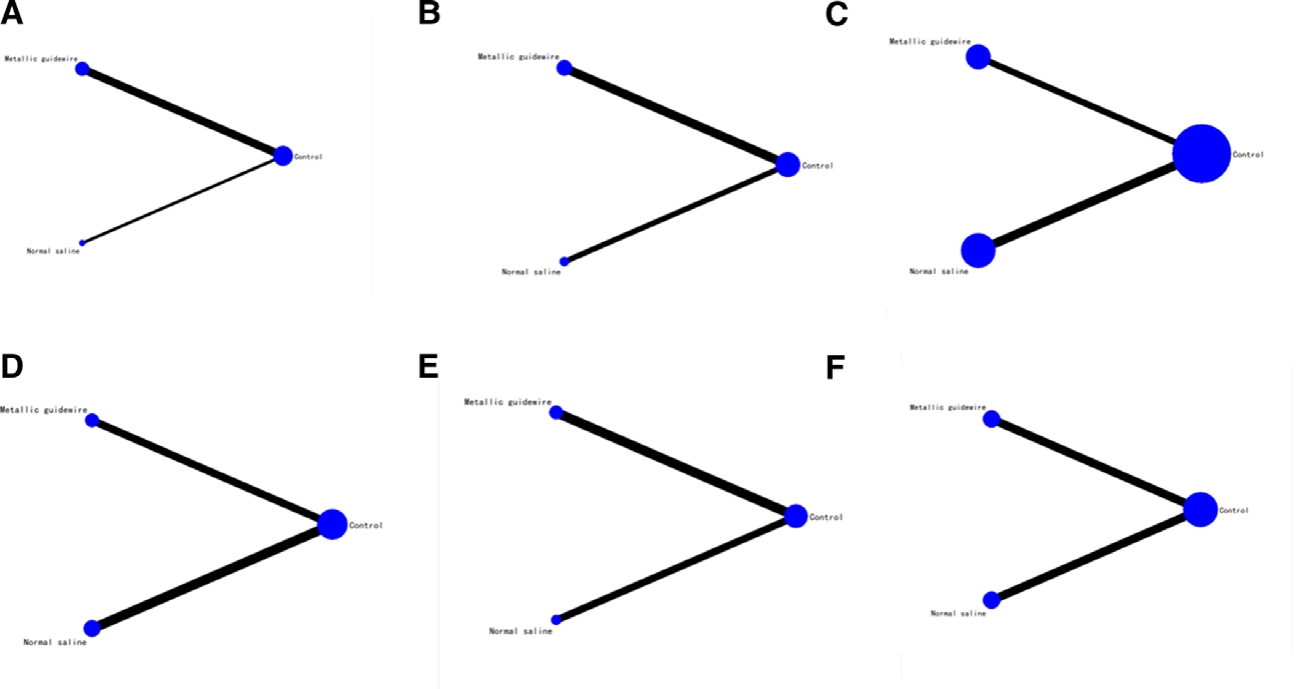
Network diagram. (A) Network diagram of the incidence of catheter-related bloodstream infections. (B) Network diagram of the incidence of thrombosis/occlusion. (C) Network diagram of the tip position accurate. (D) Network diagram of the incidence of phlebitis. (E) Network diagram of the incidence of arrhythmia. (F) Network diagram of complication.

Three interventions were involved in twenty studies, involving a total of 2566 newborns. The network diagram was drawn using Stata 14.0. The dots represent the 3 interventions, and the lines between the dots indicate a direct comparison between the 2 interventions (Fig. 16). The thicker the lines, the greater the number of studies included in that intervention.

#### 3.5.2. Results of network meta-analysis

##### 3.5.2.1. Accuracy of catheter tip position

**Figure 17.**
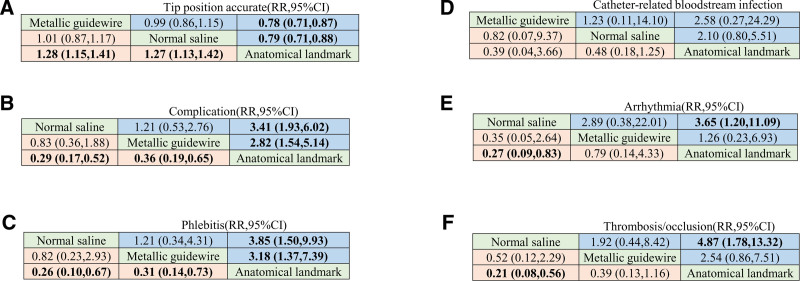
League chart.

When the 95% CI of RR contained 1, the comparison between the 2 interventions was not statistically significant. Otherwise, it was statistically significant. Compared with traditional anatomical positioning, both the metallic guidewire IC-ECG method and the normal saline column IC-ECG method can improve the accuracy of PICC catheter tip position (*P* < .05); there was no significant difference between other interventions (*P *> .05) (Fig. 17A).

##### 3.5.2.2. Incidence of complications

Compared with traditional anatomical positioning, both the metallic guidewire IC-ECG method and the normal saline column IC-ECG method can reduce the incidence of PICC catheter complications (*P* < .05); there was no significant difference between other interventions (*P* > .05) (Fig. 17B).

##### 3.5.2.3. Incidence of phlebitis

Compared with traditional anatomical positioning, both the metallic guidewire IC-ECG method and the normal saline column IC-ECG method can reduce the incidence of phlebitis (*P* < .05); there was no significant difference between other interventions (*P* > .05) (Fig. 17C).

##### 3.5.2.4. Incidence of CRBSIs

There was no significant difference between the different interventions (*P* > .05) (Fig. 17D).

##### 3.5.2.5. Incidence of arrhythmia

Compared with traditional anatomical positioning, both the metallic guidewire IC-ECG method and the normal saline column IC-ECG method can reduce the incidence of arrhythmia (*P* < .05); there was no significant difference between other interventions (*P* > .05) (Fig. 17E).

##### 3.5.2.6. Incidence of thrombosis/occlusion

Compared with traditional anatomical positioning, both the metallic guidewire IC-ECG method and the normal saline column IC-ECG method can reduce the incidence of thrombosis/occlusion (*P* < .05); there was no significant difference between other interventions (*P* > .05) (Fig. 17F).

#### 3.5.3. Sucra probability ranking

**Figure 18.**
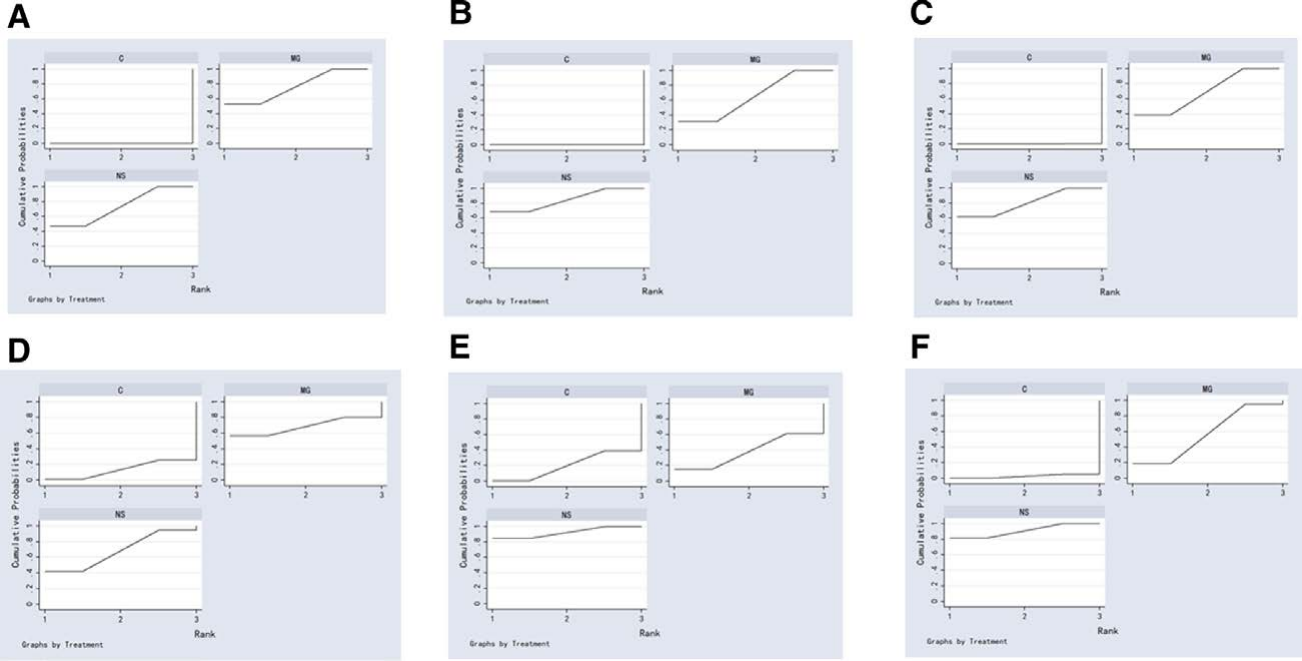
Ranking diagram. (A) Ranking diagram of the tip position accurate. (B) Ranking diagram of complication. (C) Ranking diagram of the incidence of phlebitis. (D) Ranking diagram of the incidence of catheter-related bloodstream infections. (E) Ranking diagram of the incidence of arrhythmia. (F) Ranking diagram of the incidence of thrombosis/occlusion. C = control, MG = metallic guidewire, NS = normal saline.

The cumulative probability of SUCRA for different interventions on the accuracy of location at the tip, incidence of complications, incidence of CRBSIs, incidence of phlebitis, incidence of arrhythmia and incidence of thrombosis/occlusion is shown in Figure 18. The SUCRA value represents the area under the cumulative probability curve ranging from 0 to 100. A higher value indicates a better ranking in the network analysis and a greater likelihood of becoming the optimal selection. According to the area under the curve diagram of SUCRA, the accuracy of tip positioning and the incidence of CRBSIs of the 3 kinds of interventions were ranked from best to worst as follows: metallic guidewire IC-ECG method > normal saline column IC-ECG method > traditional anatomical positioning (Table 2). Regarding incidence of complication, incidence of phlebitis, incidence of arrhythmia, and incidence of thrombosis/occlusion, the cumulative probability of SUCRA was ranked as follows: normal saline column IC-ECG method > metallic guidewire IC-ECG method > traditional anatomical positioning (Table 2).

**Table 2:** The surface under the cumulative ranking of various interventions.

Outcomes	Interventions	SUCRA
Accuracy of catheter tip position	Metallic guidewire IC-ECG method	76.40
	Normal saline column IC-ECG method	73.60
	Traditional anatomical positioning	0.00
Incidence of complications	Metallic guidewire IC-ECG method	65.80
	Normal saline column IC-ECG method	84.20
	Traditional anatomical positioning	0.00
Incidence of phlebitis	Metallic guidewire IC-ECG method	68.90
	Normal saline column IC-ECG method	80.80
	Traditional anatomical positioning	0.30
Incidence of catheter-related bloodstream infections	Metallic guidewire IC-ECG method	68.40
	Normal saline column IC-ECG method	68.30
	Traditional anatomical positioning	13.40
Incidence of arrhythmia	Metallic guidewire IC-ECG method	38.30
	Normal saline column IC-ECG method	91.90
	Traditional anatomical positioning	19.90
Incidence of thrombosis/occlusion	Metallic guidewire IC-ECG method	56.90
	Normal saline column IC-ECG method	90.60
	Traditional anatomical positioning	2.50

IC-ECG = intracavitary electrocardiogram, SUCRA = surface under the cumulative ranking.

#### 3.5.4. Publication bias and small sample effect evaluation

**Figure 19.**
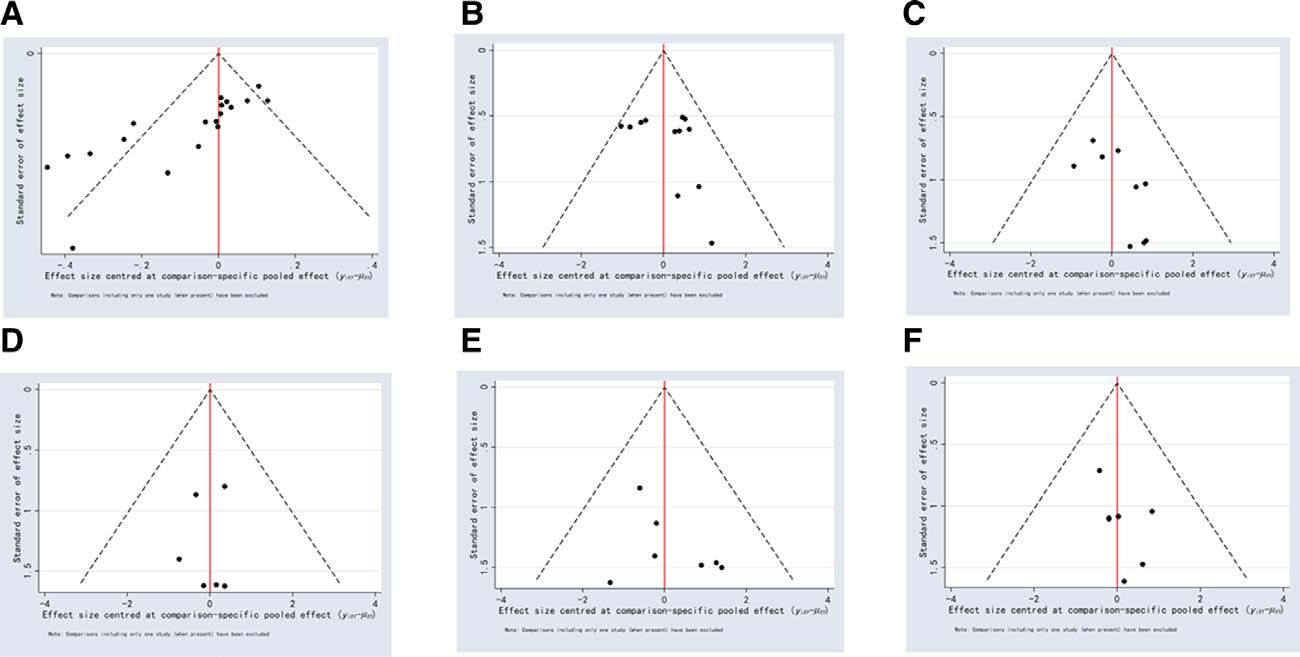
Publication bias. (A) Funnel plot of the tip position accurate. (B) Funnel plot of complication. (C) Funnel plot of the incidence of phlebitis. (D) Funnel plot of the incidence of catheter-related bloodstream infections. (E) Funnel plot of the incidence of arrhythmia. (F) Funnel plot of the incidence of thrombosis/occlusion.

Stata 14.0 was used to detect the small sample effect of each outcome indicator, and a comparison-adjusted funnel plot was drawn. The results showed that the symmetry of the funnel plot was poor, which indicated the possible existence of publication bias (Fig. 19).

## 4. Discussion

Chest radiographs were previously regarded as the gold standard for determining the precise location of PICC catheter tips.^[48,49]^ Currently, the interpretation of chest radiographs is often challenging due to the ongoing controversy surrounding radiological markers for catheterization. Simultaneously, if the catheter tip is incorrectly positioned, repeated adjustments are necessary, leading to increased radiation exposure.^[50]^ The utilization of IC-ECG can enhance the precision of PICC tip positioning.^[51]^ The potential height and variation of the P wave can effectively indicate the precise location of the PICC catheter tip.^[52]^ By analyzing the characteristic changes in ECG P-wave morphology, we can accurately determine the optimal position of the catheter and promptly identify and correct any catheter ectopic occurrences, thereby enhancing the precision of catheter tip placement.^[53,54]^ This study was to conduct a meta-analysis comparing the effectiveness and safety of metallic guidewire IC-ECG with normal saline column IC-ECG for neonatal PICC catheter tip localization.

A meta-analysis by Gan et al^[49]^ demonstrated that IC-ECG-guided PICC catheterization significantly reduced the incidence of phlebitis and complications in neonatal therapy, while also enhancing the precision of optimal tip placement. A meta-analysis conducted by Liu et al^[55]^ demonstrated that IC-ECG exhibited superior accuracy in positioning PICC tips compared to traditional X-ray methods. Furthermore, a direct comparative meta-analysis of this study revealed that both metallic guidewire IC-ECG and normal saline column IC-ECG significantly reduced the incidence of phlebitis and complications while improving the precision of optimal tip placement. Oliver et al^[56]^ demonstrated a significantly higher accuracy rate (98%) for IC-ECG in locating the catheter tip compared to X-ray localization (85%). Moreover, it is widely acknowledged in various countries, including Canada, the United States, Germany, and Italy, that routine X-ray examination is unnecessary when determining the location of the catheter tip using IC-ECG.^[48]^ The network meta-analysis results demonstrated that the complication rate and accuracy of optimal catheter tip positioning were superior to conventional methods. However, in terms of tip location accuracy and incidence of CRBSI, the metallic guidewire method proved to be the most effective. The normal saline column method exhibited the lowest performance in terms of complication rate, phlebitis rate, arrhythmia rate, and thrombosis/occlusion rate. The advantage of using normal saline column IC-ECG is that it avoids guide wire stimulation and reduces damage and inflammatory response to blood vessel walls, resulting in a lower complication rate. The normal saline column method includes manual injection, natural sagging technique, and automatic infusion pump pumping methods which may lead to variations in PICC catheter tip positioning accuracy. Therefore, based on the results from this network meta-analysis, it can be concluded that the metallic guidewire IC-ECG method outperforms the normal saline column IC-ECG method in terms of tip positioning accuracy. In future research studies, RCTs comparing different techniques within the normal saline column method should be conducted to provide further evidence for clinical application.

The utilization of the normal saline column in IC-ECG offers several advantages. Firstly, this method is highly safe, as it involves inserting a soft catheter into the vein during PICC placement, thereby minimizing vascular intimal damage that may be caused by metal guide wires. Secondly, it ensures high accuracy in catheter tip positioning. Lastly, it is cost-effective due to the widespread availability and low cost of normal saline across hospital departments. Furthermore, the guidance provided by the normal saline column technique allows for real-time adjustment of the catheter tip position during the procedure. It also supports positional adjustments in newborns with long-term indwelling PICCs, whose growth-related anatomical changes may alter the catheter tip location; such adjustments can be effectively guided using endovascular electrocardiography with normal saline.

In clinical practice, healthcare providers should consider the specific condition of the pediatric patient, the availability of equipment, and the level of expertise among medical staff when selecting an appropriate method. Generally, metallic guidewire IC-ECG demonstrates higher accuracy, whereas normal saline column IC-ECG offers greater advantages in terms of safety and ease of use. Therefore, a comprehensive evaluation is essential to determine the most suitable approach. The decision should be tailored to the individual characteristics of each patient. Moreover, future updates to clinical guidelines may provide more specific recommendations and standardized guidance.

### 4.1. Limitations of the study

This study has several limitations. Firstly, the lack of a direct comparison between metallic guidewire IC-ECG and normal saline column IC-ECG may compromise the robustness of the results. Secondly, the results of the publication bias analysis suggest the presence of potential bias, which could partially undermine the validity of the research findings. Furthermore, all RCTs included in this study were conducted in China, indicating the need for further investigation to assess the generalizability of these findings to other regions.

## 5. Conclusion

In summary, according to the network meta-analysis, preliminary evidence indicates that metallic guidewire IC-ECG may offer higher accuracy in neonatal PICC catheter tip localization compared to normal saline column IC-ECG. However, it is worth noting that normal saline column IC-ECG may carry a lower risk of complications. Nevertheless, given the limitations and potential biases of the current studies, further research is needed to confirm and substantiate these findings.

## Acknowledgments

The authors thank the authors of the included studies whonshared the important data.

## Author contributions

**Conceptualization:** Zhanglin Wang, Yan Gao, Yi Zhang.

**Data curation:** Rong Zhang, Chaojin Zhao.

**Formal analysis:** Yan Gao, Yi Zhang, Chaojin Zhao.

**Investigation:** Yunhong Lei, Rong Zhang, Chaojin Zhao.

**Methodology:** Yan Gao, Yunhong Lei, Rong Zhang, Yi Zhang, Chaojin Zhao.

**Project administration:** Yunhong Lei, Rong Zhang, Chaojin Zhao.

**Resources:** Yan Gao, Yi Zhang, Chaojin Zhao.

**Software:** Yunhong Lei, Rong Zhang, Chaojin Zhao.

**Supervision:** Yan Gao, Yi Zhang.

**Validation:** Yunhong Lei, Rong Zhang.

**Visualization:** Yunhong Lei, Rong Zhang, Chaojin Zhao.

**Writing – original draft:** Zhanglin Wang, Yan Gao, Yi Zhang.

**Writing – review & editing:** Zhanglin Wang, Yan Gao, Yi Zhang.
